# Expression Pattern of DAB Adaptor Protein 2 in Left- and Right-Side Colorectal Carcinoma

**DOI:** 10.3390/genes14071306

**Published:** 2023-06-21

**Authors:** Ivan Šustić, Anita Racetin, Katarina Vukojević, Benjamin Benzon, Ante Tonkić, Željko Šundov, Mario Puljiz, Merica Glavina Durdov, Natalija Filipović

**Affiliations:** 1 Division of Gastroenterology, Department of Internal Medicine, University Hospital of Split, Spinčićeva 1, 21000 Split, Croatia; ivan.sustic2@gmail.com (I.Š.); ante.tonkic@mefst.hr (A.T.); zsundov@gmail.com (Ž.Š.); 2 Department of Anatomy, Histology and Embryology, University of Split School of Medicine, Šoltanska 2, 21000 Split, Croatia; anitamuic10@gmail.com (A.R.); benzon.benjamin@mefst.hr (B.B.); 3 University of Split School of Medicine, Šoltanska 2, 21000 Split, Croatia; 4 Clinical Department of Gynaecologic Oncology, University Hospital for Tumours, Sestre Milosrdnice University Hospital Centre, 10000 Zagreb, Croatia; puljiz.kzt@gmail.com; 5 Department of Pathology, Forensic Medicine and Cytology, University Hospital of Split, University of Split School of Medicine, Spinčićeva 1, 21000 Split, Croatia; merigdst@yahoo.co.uk; 6 Laboratory for Experimental Neurocardiology, Department of Anatomy, Histology and Embryology, University of Split School of Medicine, Šoltanska 2, 21000 Split, Croatia

**Keywords:** colorectal cancer, anatomical subdivision, left and right colon, DAB2, DOC-2

## Abstract

Left-sided and right-sided colorectal cancer (L-CRC and R-CRC) have relatively different clinical pictures and pathophysiological backgrounds. The aim of this study was to investigate the presence of DAB adapter protein 2 (DAB2) as a potential molecular mechanism that contributes to this diversity in terms of malignancy and responses to therapy. The expression of the suppressor gene DAB2 in colon cancer has already been analyzed, but its significance has not been fully elucidated. Archived samples from 34 patients who underwent colon cancer surgery were included in this study, with 13 patients with low-grade CRC and 21 with high-grade CRC. Twenty of the tumors were R-CRC, while 14 were L-CRC. DAB2 expression was analyzed immunohistochemically in the tumor tissue and the colon resection margin was used as a control. Tumors were divided into L-CRC and R-CRC, with splenic flexure as the cutoff point for each side. The results showed that R-CRC had lower DAB2 protein expression compared to L-CRC (*p* = 0.01). High-grade tumors had reduced DAB2 expression compared to low-grade tumors (*p* = 0.02). These results are consistent with the analysis of *DAB2* gene expression data that we exported from the TCGA Colon and Rectal Cancer Study (COADREAD). In 736 samples of colon cancer, lower *DAB2* gene expression was found in R-CRC compared to L-CRC (*p* < 0.0001). *DAB2* gene expression was significantly higher in the sigmoid colon than in the cecum and ascending colon (*p* < 0.01). The analysis confirmed a lower expression of the *DAB2* in tumors with positive microsatellite instability (*p* < 0.001). In conclusion, DAB2 has a role in the biological differences between R-CRC and L-CRC and its therapeutic and diagnostic potential needs to be further examined.

## 1. Introduction

Colorectal cancer (CRC) is the third most frequent cancer and a second leading cause of death in patients with malignancies, with rising prevalence [1]. According to the World Health Organization (WHO) there are about 1.8 million cases of CRC yearly, with recorded 861,000 deaths being in 2018 [2]. Despite progress in the diagnostics and treatment of CRC, its 5-year survival is still poor, and consequently new topics in CRC pathogenesis have the potential to provide new diagnostic, prognostic and therapeutic approaches.

Over 90% of CRCs are adenocarcinomas. These are histologically classified into grade (G) 1—well and moderately differentiated and G2—poorly differentiated [3]. Around 55% of CRCs occur in the descending colon and rectum [4]. The localization of CRC plays an important role in diagnostic and therapeutic approaches. According to location, the CRC subtypes are right-sided tumors (proximal to) and left-sided tumors (distal to the splenic flexure). This subdivision arises from different embryological development, innervation and blood supply. The right-sided colon is supplied by the superior mesenteric artery and the left-sided colon and rectum are fed by the inferior mesenteric artery [5]. Further, different metabolic characteristics, bile acid exposure and molecular biology are mentioned [6]. Moreover, gene mutation, chromosomal instability, microsatellite instability (MSI) and CpG islands methylation were observed in left-sided and right-sided colon cancer, with chromosomal instability being more common in the left-sided CRC, and MSI in the right side tumors [6,7].

DAB adaptor protein 2 (DAB2), also known as disabled 2 (*Dab2*) and differentially expressed in the ovarian carcinoma 2 (*DOC-2*), is a gene that codes the clathrin-based transmembrane protein (CLASP) to regulate endocytosis, promote embryonic development and inhibit the growth of tumor cells [8]. DAB2 has three different splicing forms, and encodes three isoforms (p96-Dab2, p93-Dab2 and p67-Dab2), among which the p96-Dab2 isoform is essential for the development of visceral endoderm during mouse embryogenesis [9]. The main functional domain of the Dab2 molecule is the phosphotyrosine binding domain (PTB) of the N-terminal end. This is a highly conserved sequence and plays a variety of functional roles in endocytosis, cell mitosis, and growth factor signaling [10].

The signaling pathway of DAB2 regulates endocytosis by playing a role in cystic fibrosis transmembrane conductance regulator (CFTR) trafficking to the late endosome [9,11]. In addition, DAB2 contributes to several receptor-mediated signaling pathways and TGF-β receptor recycling. It is also required for TGF-β receptor signaling pathway and promotes the phosphorylation of the signal transducer against DPP Homolog 2 (SMAD2) [12]. DAB2 also mediates TFG-β-stimulated Janus kinase (JNK) activation [13,14]. Furthermore, DAB2 may interfere with the canonical Wnt/β-catenin signaling pathway by stabilizing the β-catenin destruction complex through a competing link with axin, blocking its dephosphorylation by protein phosphatase 1 (PP1) and thus inhibiting this pathway [10,15]. In addition, DAB2 inhibits cell surface growth factor/Ras signaling pathways and ERK activation by breaking the binding of GRB2 to SOS1. DAB2 inhibits SRC by activating phosphorylation at ‘Tyr-419’ [15]. DAB2 is involved in the modulation of androgen receptor (AR) signaling and competes with AR for interaction with SRC [16]. Moreover, DAB2 has a role in the CSF-1 signal transduction pathway and plays important role in cell differentiation and maturation [17]. 

During embryonic development, DAB2 has an active role in cell positioning and in forming a visceral endoderm (VE), being required in the VE response to Nodal signaling from the epiblast [17]. DAB 2 has a role in epithelial-to-mesenchymal transition, a process necessary for the proper development of normal structures, and also in malignant transformation. DAB2 is involved in myeloid cell differentiation and can induce macrophage adhesion and spreading [17]. Concerning all mentioned roles, DAB2 was intensely investigated as a potential tumor suppressor. Previous studies established a decrease in DAB2 expression as a hallmark in a wide range of human tumors, including ovarian, breast, lung and colon cancer, and choriocarcinoma [18,19,20]. 

Several studies investigated the role of DAB2 and its interactive protein (DAB2IP) in colorectal carcinoma. Son et al., 2018, [21] established that DAB2IP is lost in 20% of MSI-H colorectal tumors due to frameshift mutations. Xiao et al., 2019, [22] found a negative correlation between miRNA miR-889 and DAB2IP and concluded that miR-889 directly targets DAB2IP to accelerate tumor growth. Recently, Li et al. [23] established a negative correlation between DAB2IP and *c-MYC* protooncogene expression in colorectal cancer stem cell-like colon tumor-repopulating cells (TRC). Another described mechanism of DAB2 silencing was promoter hypermethylation. Zhou et al. discovered that DNA methyltransferase 3A (DNMT3A) activates MEK/ERK signaling pathway and stimulates CRC progression through DAB2IP inactivation [24]. Zhu et al., 2017., discovered a mechanism of DAB2IP negative effects on matrix metalloproteinase 2 synthesis and negative impacts on CRC metastasis [25]. DAB2 acts as a tumor suppressor gene using Wnt, MAPK and TGF β pathways. TGF β can be pro-tumorigenic. This mainly occurs in the prostate and anti-tumorigenic conditions, thus showing DAB2’s controversial role [26]. Despite extensive research into DAB2 in CRC, there is no study that compares the expression of DAB2 as one of the possible factors that differentiates left-sided versus right-sided tumors. Hence, the aim of our study is to evaluate expression of DAB2 in right- and left-sided CRC and correlate it with pathological, clinical and laboratory data. 

## 2. Materials and Methods

### 2.1. Normal and Carcinogenic Adult Human Tissues

The study included archived samples from 34 patients who underwent surgery for colorectal cancer in the period January–December 2017. Paraffin blocks of tumor tissue and the resection margin were collected from the archive of the Department of Pathology, Forensic Medicine and Cytology, University Hospital Split. Exclusion criteria included insufficient material in paraffin block, positive resection margin and incomplete clinical data in the hospital records.

The sample contained specimens from 13 patients with low-grade and 21 patients with high-grade colon cancer. Tumor tissue and colon resection margin, as the controls, were immunohistochemically analyzed to DAB2 expression. Patients were divided into left- (N = 14) and right-sided (N = 20) colon tumors, with splenic flexure acting as a cut-off point for each side. Ethics Committee of the University Hospital Split approved the study. 

### 2.2. Sample Preparation and Immunohistochemistry

Paraffin blocks were cut into 5 µm-thick slices. Deparaffinization was performed in xylene and tissue rehydration in decreasing grades of alcohol. Afterwards, slides were washed in distilled water. Antigen retrieval was performed by heating the sections for 12 min in a citrate buffer (pH 6.5) in a microwave oven and subsequently washed in phosphate-buffered saline (PBS). Rabbit polyclonal anti-DAB2 antibody ab33441 (Abcam, Cambridge, CB4 OFL, UK) was applied, diluted at 1:400, and left overnight at room temperature in a humidified chamber. This antibody was raised against a synthetic peptide corresponding to human DAB2 aa 750 at the C-terminus conjugated to keyhole limpet haemocyanin and was predicted to cross-react with both isoforms 1 and 2 of human DAB2. Next-day slides were washed in PBS, and the secondary antibody (AlexaFluor^®^488 Affini Pure Donkey Anti-Rabbit IgG (H + L); Jackson Immuno Research Laboratories, Inc., Baltimore, PA, USA) was applied and incubated for 60 min. After being washed in PBS, 4′,6-diamidino- 2-phenylindole (DAPI) was applied to stain nuclei, and slides were washed again and cover-slipped. 

### 2.3. Data Acquisition and Analysis

The slides were analyzed and photographed using a DS-Ri2 digital camera (Nikon, Tokyo, Japan) equipped with the NIS-Elements F software (Nikon, Tokyo, Japan). Pictures were taken in a Tiff format for further analysis. For each patient, 8 fields of tumor and 8 of resection margin were captured under 40x objective magnification. Image J software (National Institutes of Health, Bethesda, MD, USA) was applied for the processing and analysis of photomicrographs. The processing protocol was adjusted from that already published [27,28]. Initially, the fluorescence leakage reduction was made by the subtraction of red counter-signals from green fluorescence and subsequently the median filter was used with a radius of 5.0 pixels. Then, the “default” thresholding algorithm method was applied. Analysis was performed by measuring the fluorescence percentage area. The percentage of DAB2 expression in tumor and the healthy resection margin of each patient were calculated to compare eventual changes. 

### 2.4. Statistical Analysis

Box–Cox transformation of data was carried out in order to satisfy normality presumption in *t*-test and multivariate model. Raw data were presented in tables and graphs. Univariate analysis was performed by *t* test and determining the Pearson correlation between relative DAB2 expression and clinic-pathological variables. Multivariate analysis was performed by multiple linear regression modelling. Statistical evidence is presented as size effect, 95% CI of size effect, goodness of fit (R^2^) measure, ΔcAIC, and *p* values. For graphical presentation, we used the percentage of tumor DAB2 expression in comparison to the normal tissue of the same patient in order to make the results clearer for the reader.

### 2.5. Statistical Analysis in the TCGA-KIRC Cohort

In addition, we used the data for RNA expression of the *DAB2* gene exported from the UCSC Xena database (College of California Santa Cruz, Santa Cruz, CA, USA) [29]. We used TCGA colon and rectum adenocarcinoma (COADREAD) gene expression by RNAseq (polyA+ IlluminaHiSeq). The dataset is combined from TCGA colon adenocarcinoma and rectum adenocarcinoma datasets. The gene expression profile was measured experimentally using the Illumina HiSeq 2000 RNA Sequencing platform released by the University of North Carolina TCGA genome characterization center. Level 3 data were downloaded from the TCGA data coordination center. This dataset shows the gene-level transcription estimates, as in log2(x + 1)-transformed RSEM normalized counts. Genes are mapped onto the human genome coordinates using UCSC Xena HUGO probeMap [29]. Data from the TCGA Colon and Rectal Cancer (COADREAD) study, which contained 736 colon and rectal cancer specimens with no history of neoadjuvant treatment, were obtained, exported as text, and imported into an Excel data sheet. We removed samples that did not contain data for *DAB2* expression, and 733 samples remained for analysis. Only 413 samples contained data about the anatomical location, of which 193 were R-CRC (82 were located in cecum, 62 in ascending colon, 21 in hepatic flexure and 25 in transverse colon) and 220 were L-CRC (7 in splenic flexure, 15 in descending colon, 100 in sigmoid colon, 52 at rectosigmoid juncture and 46 in rectum). The results obtained were compared using one-way ANOVA, followed by Tukey’s multiple comparisons test, while a comparison of right- and left-sided tumors was made via an unpaired *t* test. Prior to exportation, we used UCSC Xena database integrated statistical software (College of California Santa Cruz) [29] to compare *DAB2* gene expression according to: primary lymph node presentation assessment, perineural invasion present, pathologic stage, pathologic T, pathologic N, pathologic M, number of lymph nodes positive by haematoxylin-eosin staining, number of first degree relatives with cancer diagnosis, microsatellite instability, lymphatic invasion, *K-RAS* mutation found, and gender and age at initial pathologic diagnosis. Only microsatellite instability has shown a relation with *DAB2* gene expression. Hence, we compared exported data for *DAB2* expression in microsatellite stable and unstable CRC tumors by using an unpaired *t* test. 

## 3. Results

**Figure 1:**
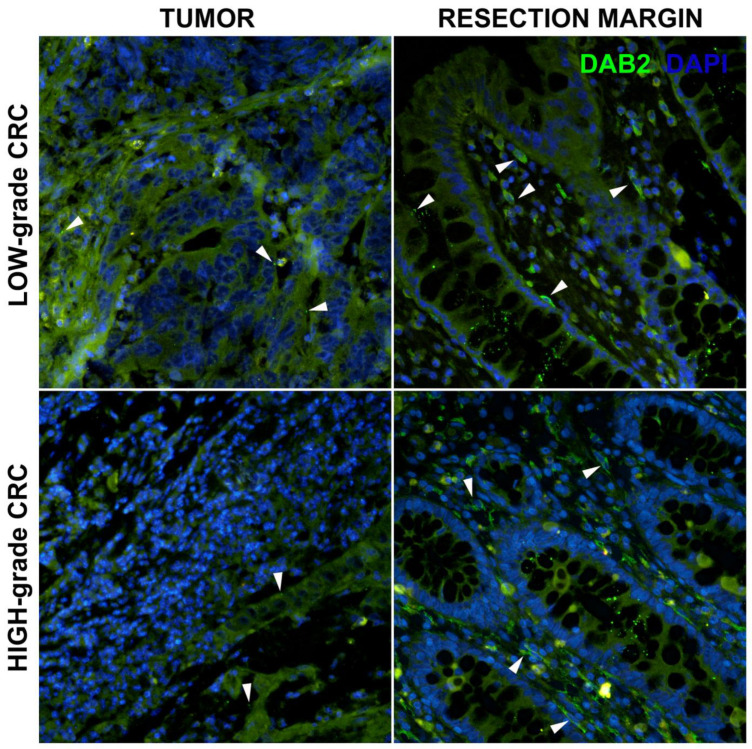
Expression of DAB2 (green) in low- and high-grade colorectal tumor (CRC) and colon resection margin of the same patient. Strong DAB2 expression was found in spindle-shaped cells of the *lamina propria* and apical part of the goblet cells in the epithelium of the normal mucosa (arrowheads). Residual weak expression was occasionally observed in tumor tissue (arrowheads). Blue—DAPI.

**Figure 2:**
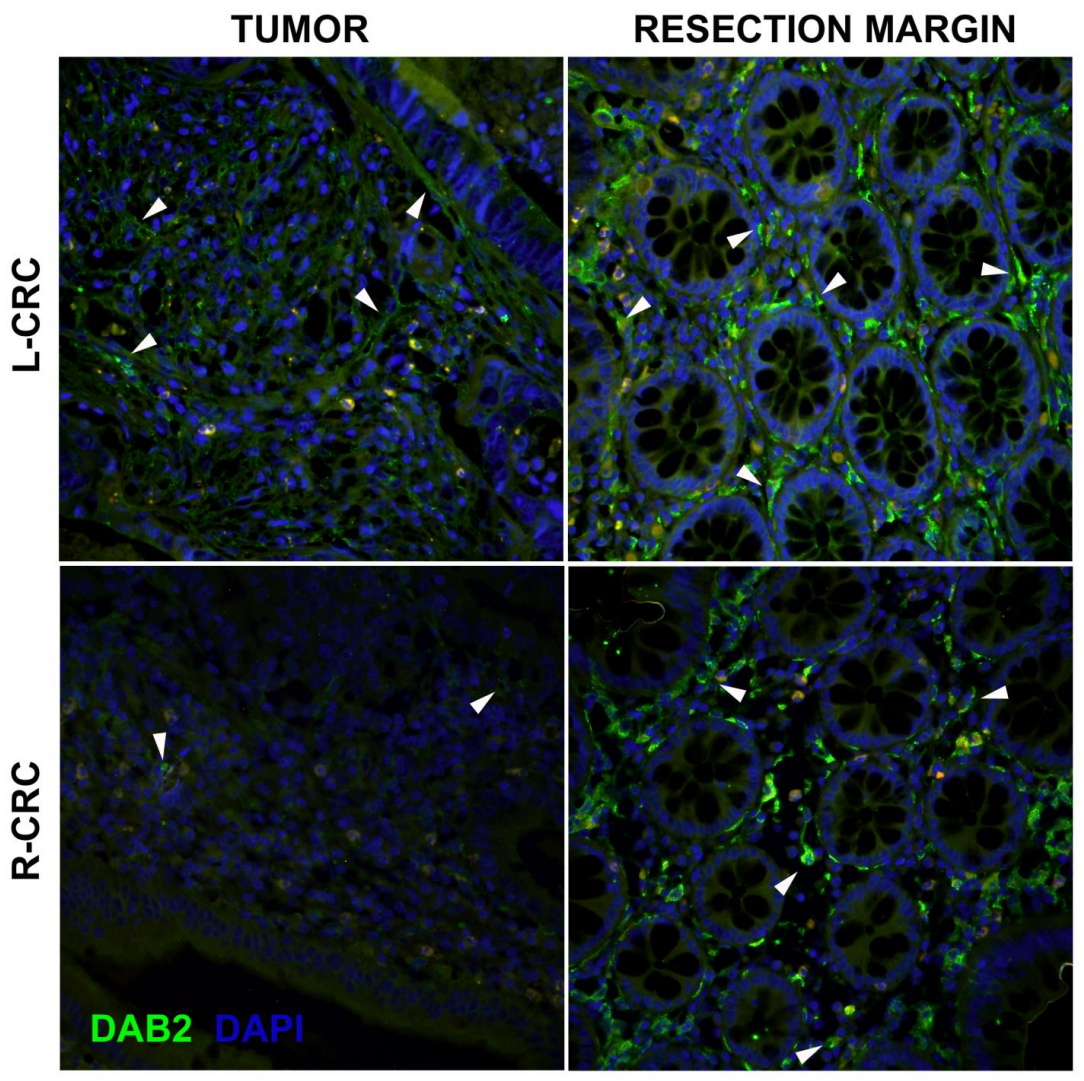
Expression of DAB2 (green) in left- and right-sided colorectal tumor (L-CRC and R-CRC, respectively) and colon resection margin of the same patients. Strong DAB2 expression was found in spindle-shaped cells of the *lamina propria* and apical part of the goblet cells in the epithelium of the normal mucosa (arrowheads). Residual weak expression was occasionally observed in tumor tissue (arrowheads)—more DAB2 expression was observed in L-CRC, in comparison to the R-CRC. Blue—DAPI.

Immunohistochemistry was performed to explore DAB2 expression in terms of the normal colon resection margin and corresponding colorectal adenocarcinoma. DAB2 expression within a healthy resection margin was mostly observed in spindle cells of the *lamina propria* and at the apical side of the goblet epithelial cells (Figure 1 and Figure 2). In low-grade and high-grade CRC, a diminishing expression of DAB2 was observed, and this rarely presented in tumor cells or stroma (Figure 1). In addition, more DAB2 expression was observed in L-CRC in comparison to the R-CRC (Figure 2).

In our cohort, 20 patients had R-CRC and 14 had L-CRC. According to univariate analysis, R-CRC have had a more decreased expression of DAB2 protein when compared with L-CRC (*p* = 0.01; Table 1). Furthermore, high-grade tumors had DAB2 expression that decreased more than that of low-grade tumors (*p* = 0.02). Multivariate analysis was performed to determine whether location and histological grade are independent predictors of DAB2-relative expression (Table 2). DAB2 expression difference decreased by 52.21% in L-CRC. In high-grade tumors, DAB2-relative expression was 45.9% different from expression in normal healthy intestinal margin of the same patient.

**Figure 3:**
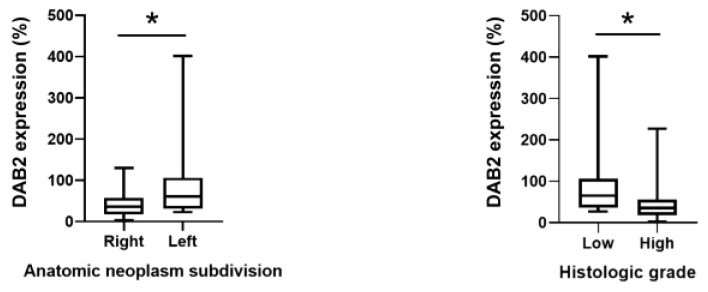
DAB2 expression in CRC (percentage from expression in normal mucosa on colon resection margin of each patient), according to anatomical subdivision and histologic grade. * *p* < 0.05 between indicated groups.

DAB2 expression in CRC (expressed as percentage of expression in normal healthy intestinal margin of the same patient) was significantly lower in R-CRC than L-CRC (*p* < 0.05; Figure 3). DAB2 expression was significantly lower in high-grade CRC than in low-grade CRC (*p* < 0.05; Figure 3).

**Figure 4:**
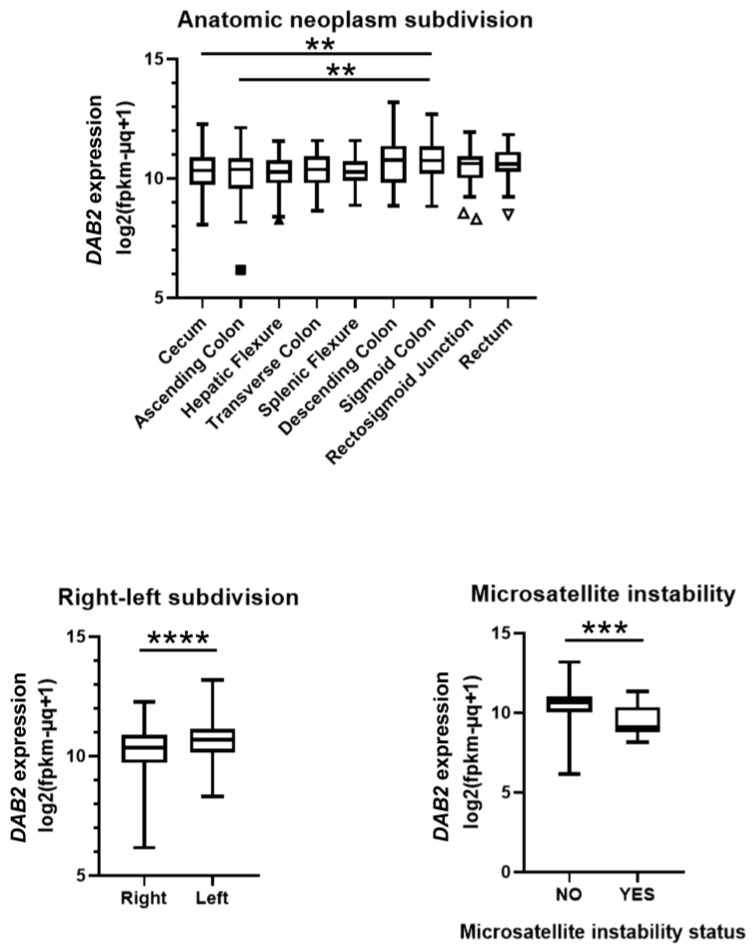
From UCSC Xena database (University of California Santa Cruz, Santa Cruz, CA, USA) (Goldman et al. [29]) we exported data for RNA expression of DAB2. Study TCGA Colon and Rectal Cancer (COADREAD) contained data of 736 colorectal cancer samples. Out of them, 733 had data of DAB2 expression. 413 samples contained data about the anatomical location. The analysis prior the exportation has shown relation of microsatellite instability with DAB2 gene expression. RNA expression is presented as log2(x + 1)-transformed RSEM normalized count. ** *p* < 0.01; *** *p* < 0.001; **** *p* < 0.0001 between indicated groups.

The analysis of DAB2 gene expression data exported from the TCGA Colon and Rectal Cancer (COADREAD), which contained 736 colorectal cancer samples, has found lower DAB2 gene expression in R-CRC, in comparison to the L-CRC (*p* < 0.0001; Figure 4). In particular, DAB2 gene expression was significantly higher in tumors located in the sigmoid colon than in the caecum and ascending colon (*p* < 0.01, both; Figure 4). Preliminary analysis did not find a correlation between DAB2 expression and status of lymph nodes, perineural invasion, pathologic stage (pT, pN, pM), number of positive lymph nodes, lymphatic invasion, number of first-degree relatives with cancer diagnosis, K-RAS mutation, and gender and age at initial diagnosis (data were not shown). On the one hand, MSI was significantly connected to DAB2 gene expression. Our analysis confirmed lower DAB2 gene expression in that were tumors positive for MSI (*p* < 0.001; Figure 4). In our cohort, only three patients had tumors that were MSI-instable and all of them were R-CRC. This number was too small for statistical comparison; however, mean expression of DAB2 was lower in MSI-instable (YES; 41.37%; N = 3) in comparison to the MSI-stable (NO; 66.56; N = 31) tumors. On the other hand, in the COADREAD study, out of 114 tumors for which both anatomical location and MSI status were known, 51 were R-CRC and 10 of them had MSI-YES status (19.61%), while 63 were L-CRC, and only one had MSI-YES status (1.59%).

## 4. Discussion

Apart from the growing evidence of biological and pathophysiological differences between R-CRC and L-CRC, all molecular mechanisms of their different behavior in terms of malignancy and response to therapy remain to be discovered. Tumors on the right side are more difficult to diagnose and become symptomatic in an advanced stage. Therefore, there is a need for the further refinement of the diagnostic and therapeutic approach. Although DAB2 has long been known as a tumor suppressor in colon cancer, its predictive and prognostic potential is still insufficiently investigated. Previous studies pointed out the potential of *DAB2* as a tumor suppressor gene since a decrease in *DAB2* expression was observed in a wide range of tumors, [18,19,20]. However, role for DAB2 protein in colon cancer is still not completely understood and further research is needed to prove its tumor suppressor potential. Since CRC is a very common malignancy, with high mortality and growing incidence [2], novel biomarkers are needed to determine the best prognostic and therapeutic approach to tke [30,31].

There is a growing body of data on biological and pathophysiological differences of CRC according to localization. Besides embryological, vascular/nerve supply and metabolic differences, it was known for a while that right and left CRC are also different in their genetic mechanisms of carcinogenesis: according to the literature, R-CRC are more often related to a MSI, while L-CRC are more often characterized by CIN [4,21,32]. In addition, the CpG island methylator phenotype (CIMP), which occurs in serrated-pathway tumorigenesis of colon cancer, is more common in right-sided tumors and is often accompanied by *BRAF* and *TP53* mutation profile [33]. 

We hypothesized that DAB2 protein might be differently expressed in R-CRC and L-CRC and that change in its expression might have prognostic relevance. Hence, we explored the expression of DAB2 in CRC by using immunohistochemistry. The results of our study were in agreement with the results of Kleeff et al., who established the down-regulation of DAB2 in colon cancer [18], focusing on CRC as a single entity. We found a decrease in DAB2 expression in most of the CRC tumor specimens in comparison with the healthy intestinal margin extracted from the same patients. However, in some patients, DAB2 expression in CRC even increased severalfold. In addition, we found a substantially larger decrease in DAB2 expression in the R-CRC, in comparison to the L-CRC, in which higher expression was observed. 

The loss of DAB2 expression in the R-CRC might be related to its role in TGF β signaling. This signaling pathway is important in colon cancer development, especially in the right colon [6]. In early tumorigenesis, DAB2 promotes the TGF β/SMAD pathway, supressing tumor growth and promoting epithelial mesenchymal transition [34]. The TGF-β pathway is inactivated in dMMR/MSI-high tumors, which are also more often found in the right colon [6]. One of the explanations for lower DAB2 expression in R-CRC might be the silencing of DAB2 through hypermethylation [34], which is a more characteristic mechanism of tumorigenesis in the right colon. This type of tumorigenesis is common for serrated pathways and sessile serrated adenomas, which are characteristically located in the right side of the colon [35]. However, future studies are needed to confirm this hypothesis.

The results that we obtained by using immunohistochemistry were confirmed by an analysis of the publicly available *DAB2* transcriptome from the TCGA Colon and Rectal Cancer (COADREAD) study. The expression of the *DAB2* gene was significantly lower in R-CRC. It was the highest in tumors of the sigmoid colon, and lowest in tumors from the cecum and the ascending colon. We found a correlation of *DAB2* gene expression and MSI. Significantly lower *DAB2* gene expression was found in tumors with positive MSI. Altogether, these data are in agreement with the fact that MSI is more frequent in R-CRC [21]. These data are in agreement with the results of our study: in our cohort, only three (out of 34 patients) had MSI unstable tumors, and all of them were R-CRC. In addition, analysis of data from COADREAD study specimens confirmed that 51 patients had R-CRC and 10 of them had MSI-YES status (19.61%), while 63 patients had L-CRC and only one of them had MSI-YES status (1.59%). It is interesting that *DAB2* mutations were also found to be related to MSI. According to Son and collaborators, *DAB2* shift mutations were found in 2 of 79 CRCs (2.5%) with MSI-H, and they were not detected in MSS tumors [21]. 

We also found that the decrease in DAB2 protein expression was significantly greater in high-grade than low-grade CRC. This is consistent with the previously proposed role of DAB2 as a tumor suppressor. DAB2 was initially known for its role in ovarian cancer [20]. Later, DAB2 overexpression was found to directly inhibit prostate cancer cell proliferation by down-regulating AKT and ERK kinases [36]. Ma et al. also found that lower DAB2 expression correlated with higher TNM status, more frequent metastases, and worse outcomes in non-small-cell lung cancer, and suggested that DAB2 could become a new prognostic and predictive biomarker, especially in DAB2 hypermethylated tumors [37].

The tumor suppressor role of DAB2 in CRC might be related to the activity of DAB2 in the uptake of vitamin D receptor binding protein (DBP) in colon and prostate cells [36], a topic that concerns the role of vitamin D as a tumor suppressor in the colon, and other organs [37]. Hence, a decrease in DAB2 expression might result in disturbed vitamin D trafficking in epithelial cells, which results in higher susceptibility to malignant transformation. In addition, DAB2 is an inhibitor of the Wnt-β catenin pathway. Hocevar et al. found that DAB2 inhibits Wnt signaling pathway through dishevelled–axin interaction [13]. This interaction sends a signal to another tumor suppressor in colon “adenomatous polyposis coli” (APC), axin, and glycogen synthase kinase 3b and β catenin, leading to the phosphorylation of β catenin on a N terminal and destruction through the ubiquitin pathway, consequently promoting cell proliferation [13]. Moreover, already mentioned role of DAB2 in TGF β signaling has important potential as a tumor suppressor. Hannigan et al. found that DAB2 promotes TGF β/SMAD pathway in early tumorigenesis and that this supresses tumor growth [34]. They also found down-regulated DAB2 in advanced cancer with poor prognosis, in which TGF β function was changed from that of the tumor suppressor to tumor promoter. This is in agreement with our finding of low DAB2 expression in high-grade CRC. 

Differences in DAB2 expression in right- and left-sided CRC may be associated with predictive factors. Patients with L-CRC tumors benefit more from targeted therapies (including anti-EGFR or anti-VEGFR antibodies), while patients with MSI-high R-CRC tumors benefit more from immunotherapies [6]. Recent studies have suggested that miR-93 promotes prostate cancer progression/metastasis by suppressing DAB2 to activate the Akt/ERK1/2 pathway, and that the upregulation of DAB2 and inactivation of Akt/ERK1/2 may be a potential therapeutic target for prostate cancer green tea polysaccharide [36]. Furthermore, it has been shown that X radiation can induce DAB2 in hypermethylated non-small-cell lung cancer by up-regulating DNA methyl transferase (DNMT), the enzyme that demethylates the promoter region of *DAB2* [37]. In our study, *DAB2* was down-regulated in high-grade CRC tumors. One of the mechanisms of DAB2 silencing is methylation in the promoter region, and therapeutic potential of this fact remains to be explored [33]. 

The main drawback of the present study is that we only provided data for our cohort about the DAB2 protein expression form immunohistochemistry. We used immunohistochemistry to reveal the spatial distribution of the DAB2 protein in different cells, but also to quantify DAB2 protein expression. Nevertheless, our results were strongly supported by data on *DAB2* RNA expression on a large number of patients from the COADREAD study. Hence, we believe that the complimentary approach used in our study contributes to the knowledge of the role of DAB2 in CRC and supports previous findings on its tumor suppressor role.

DAB2 is an endocytic adaptor protein that is implicated in clathrin-mediated endocytosis. DAB2 was initially characterized as a protein with two alternatively spliced forms: p96 and p67, with the later lacking a central exon. The form p67 is less efficient in endocytosis and does not associate with clathrin [38]. In our study, we raised an antibody against a synthetic peptide. This corresponded to human DAB2 aa 750 at the C-terminus that was predicted to cross-react with two isoforms, namely, 1 and 2, of human DAB2. Hence, we cannot distinguish between two different isoforms in this case. Further studies are needed to reveal the particular role played by DAB2 isoforms in high-/low-grade CRC and explain their potential roles in R-CRC and L-CRC pathogenesis.

## 5. Conclusions

We found that DAB2 expression was lower in R-CRC than L-CRC, and greater in high-grade CRC than om low-grade CRC. The down-regulation of *DAB2* expression is associated with MSI, which is characteristic of R-CRC. The possibility of the pharmacological modulation of *DAB2* gene expression and/or DAB2 function in human cancer might provide an opportunity to suppress the proliferation of malignant cells. These intriguing findings deserve further investigation in order to shed light on the role of DAB2 in CRC pathophysiology, their associations with MSI, and their promising predictive potential.

## Figures and Tables

**Table 1:** Univariate analysis of DAB2 expression.

Variable	Pearsons r	*p* Value
Location (R-CRC/L-CRC)	−0.40475	0.017577
Histological grade	0.37953	0.026839

**Table 2:** Multivariate analysis of relative DAB2 expression.

Variables in Model	Effect Size (95% CI)	R^2^	ΔcAIC *	*p* Value *
Intercept	35.54 (−76.15 to 147.2)	28.52%	6.4	0.0055
Location (R/L)	−52.51 (−101.1 to −3.927)			
Histological grade	45.9 (−3.304 to 95.10)			

* comparison with null (intercept only) model.

## Data Availability

Data from this study are available upon reasonable request from the corresponding author.
